# Knowledge of cytology results affects the performance of colposcopy: a crossover study

**DOI:** 10.1186/s12905-024-03025-y

**Published:** 2024-03-21

**Authors:** Eva Lalande, Holly Clarke, Manuela Undurraga, Vu Quoc Huy Nguyen, Cyril Jaksic, Frederic Goffin, Marc Arbyn, Jose Jeronimo, Jean-Christophe Tille, Essia Saiji, Pierre Vassilakos, Patrick Petignat

**Affiliations:** 1 Gynecology Division, Department of Pediatrics, Gynecology and Obstetrics, University Hospitals of Geneva, Geneva, Switzerland; 2 Faculty of Medicine, University of Geneva, Geneva, Switzerland; 3 Department of Obstetrics and Gynecology, Hue University of Medicine and Pharmacy, Hué, Vietnam; 4 Clinical Research Center, Faculty of Medicine, Geneva University Hospitals, University of Geneva, Geneva, Switzerland; 5 Department of Obstetrics and Gynecology, University of Liege, Liege, Belgium; 6 Unit of Cancer Epidemiology, Belgian Cancer Centre, Sciensano, Brussels, Belgium; 7 Department of Human Structure and Repair, Faculty of Medicine and Health Sciences, University Ghent, Ghent, Belgium; 8 National Cancer Institute, Bethesda, MD USA; 9 Diagnostic Department, Division of clinical pathology, Geneva University Hospitals, Geneva, Switzerland; 10 Geneva Foundation for Medical Education and Research, Geneva, Switzerland

**Keywords:** Cervical intraepithelial neoplasia (CIN), Cytology, Colposcopy, Diagnosis, Cross-over studies

## Abstract

**Objective:**

To determine whether knowledge of cytology affects the colposcopist’s diagnostic accuracy in the identification of cervical intraepithelial neoplasia grade 2 and worse (≥ CIN2).

**Method:**

In this cross-over study, healthcare professionals interpreted colposcopy images from 80 patient cases with known histological diagnoses. For each case, 2 images taken with a colposcope were provided (native and after acetic acid application). Inclusion criteria consisted of women with a transformation zone type 1 or 2, who had both a cytological and histological diagnosis. Cases were distributed across two online surveys, one including and one omitting the cytology. A wash-out period of six weeks between surveys was implemented. Colposcopists were asked to give their diagnosis for each case as < CIN2 or ≥ CIN2 on both assessments. Statistical analysis was conducted to compare the two interpretations.

**Results:**

Knowledge of cytology significantly improved the sensitivity when interpreting colposcopic images, from 51.1% [95%CI: 39.3 to 62.8] to 63.7% [95%CI: 52.1 to 73.9] and improved the specificity from 63.5% [95%CI: 52.3 to 73.5] to 76.6% [95%CI: 67.2 to 84.0]. Sensitivity was higher by 38.6% when a high-grade cytology (ASC-H, HSIL, AGC) was communicated compared to a low-grade cytology (inflammation, ASC-US, LSIL). Specificity was higher by 31% when a low-grade cytology was communicated compared to a high-grade.

**Conclusions:**

Our data suggests that knowledge of cytology increases sensitivity and specificity for diagnosis of ≥ CIN2 lesions at colposcopy. Association between cytology and histology may have contributed to the findings.

## Introduction

Cervical cancer affects a large number of women worldwide, and represents the fourth most frequent cancer diagnosed in this population [1, 2]. In recent years, the incidence of cervical cancer in high-income countries has significantly declined, mainly due to effective prevention through vaccination, screening, diagnosis and treatment of pre-cancerous lesions [1]. Cytological screening contributes to early diagnosis of cervical precancerous lesions. When combined with colposcopy for women with an abnormal screen, it has been shown to be an effective standard for ≥ CIN2 (≥ cervical intra-epithelial neoplasia grade 2) diagnosis [3].

The colposcopy exam includes the observation of the native cervix as well as the cervix after application of 3–5% acetic acid solution to determine the presence of acetowhite lesions. Colposcopy allows localization of the lesion(s), evaluation of the lesion(s) severity, and facilitates directed biopsy for diagnosis. Results from this exam determine whether patients require biopsy, or if they can be managed conservatively with follow-up. In countries with screening programs, gynecologists may carry out colposcopies in diverse settings such as primary care, regional and tertiary hospitals. Some perform exams sporadically whilst others see large volumes, frequently in colposcopy clinics. Therefore, there is likely to be a great variability in the experience and training of colposcopists [4]. The diagnostic performance of colposcopy depends on the experience of the observers, and their skill in recognizing the acetowhitening of the epithelium after acetic acid application (thickness, color, border irregularity). Inconsistencies in performance for colposcopy have been demonstrated in the literature [5, 6] particularly for the detection of high-grade abnormalities [7]. Reported sensitivity varies from 58 to 99% [8–13] while specificity ranges from 23 to 93% [8–13] depending also on the grade of the lesions. This range of values demonstrates the inherent subjectivity of this method, limited by the clinician’s capacity to distinguish lesions, appreciate their characteristics [6] and differentiate precancerous from benign appearances, leading in some cases to a misdiagnosis. To overcome the weakness of colposcopy, investigators have suggested systematically performing cervical biopsy even if the cervix appears normal. One study demonstrated that the sensitivity for detecting ≥ CIN2 increased from 61% (95%CI: 55–67) in a single biopsy to 86% (95%CI: 80–90) with two biopsies and to 96% (95%CI: 91–99) with three biopsies [14].

Colposcopy is generally conducted with knowledge of the cytology results, but it is still unclear if the sensitivity and specificity of colposcopy is significantly impacted when interpreted in the presence of the cytological result. Alongside cytology, colposcopy may also be influenced by the knowledge of other results or demographic parameters such as HPV status, age or prior histopathology [15].

Our study’s aim was to determine whether knowledge of cytology influences the colposcopic diagnosis of ≥ CIN2 lesions.

## Method

### Case selection

**Fig. 1:**
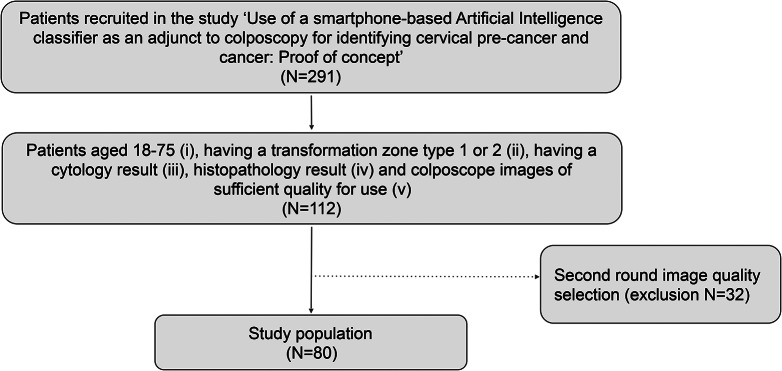
Flowchart of participants recruited for the online surveys

A total of 80 cases were collected from a cohort of women already recruited to a study entitled ‘Use of a smartphone-based Artificial Intelligence classifier as an adjunct to colposcopy for identifying cervical pre-cancer and cancer: Proof of concept’ (study number 2020 − 00868) Fig. 1. Patients were consented for this aforementioned study at the colposcopy clinic, gynecologic division of the University Hospital of Geneva, between June 2021 and June 2022, and all patients signed an informed consent. Inclusion criteria were women (i) aged 18–75, (ii) having a transformation zone type 1 or 2 and having (iii) a cytology result (iv), histopathology result and (v) colposcope images of sufficient quality for use. All cases addressed in our colposcopy setting that fulfilled inclusion criteria were considered in a chronological manner.

### Case interpretation

The study relied on specialists in colposcopy analyzing two images for each of the 80 cases, one native and one after acetic acid application. The interpretation was recorded in the form of a JotForm web-based survey. The same images were analyzed twice on two separate sessions with at least a six-week interval between the two sessions. In one of the sessions the images without cytology were analysed, while in another session the images were complemented by the cytological status. Patients were initially referred in the context of primary cytology screening, and participating colposcopists had no underlying knowledge of HPV results or other information such as the age of the patient. Each participant was asked to categorize the image as “<CIN2” or “≥CIN 2”. Overall, the study involved 40 patients with < CIN2 histopathology results, and 40 patients with ≥ CIN2 histopathology, but participants were unaware of the ratio of positive and negative cases.

### Colposcopists and interpretation rounds

A web-based Lime-survey link was distributed to multiple gynecology departments, inviting colposcopists to enroll and participate in the study. Initially, 71 participants enlisted, and ultimately, 38 of them completed both surveys and were divided into groups A and B. In keeping with the crossover design, group A received the survey without the cytology result, and then the survey with the cytology result six weeks later. Group B received the surveys in the opposite order. The participants were not aware of each other’s responses. A 6-week washout period was chosen as in the literature this has been demonstrated to confer a reduced rate of residuary recall, thus minimizing risks associated with intra-observer studies [16]. Empirically, multiple studies assessing whole-slide imaging (WSI) in pathology have used a minimum of 3 weeks as a washout period in their method [17, 18].

### Reference standard

The histological assessment of biopsy constituted the reference standard diagnosis. Patients with a ≥ CIN2 diagnosis based on biopsy underwent confirmation of the diagnosis through cone biopsy. Colposcopy-directed biopsies from all suspicious areas on examination had been undertaken for all women with histology that was revealed to be < CIN2. <CIN2 patients also had a colposcopy follow-up visit at 6 months with cytology +/-biopsy if lesions were seen. During the colposcopy examination, native images of the cervix and images after application of acetic acid were taken. All patient data was gathered retrospectively.

### Cytological and histological interpretation

The cytological results were classified according to the Bethesda system 2015 [19]. Namely: negative for intraepithelial lesions or malignancy; atypical squamous cells of undetermined significance (ASC-US); atypical squamous cells of undetermined significance that cannot exclude high-grade squamous intraepithelial lesions (ASC-H); low-grade squamous intraepithelial lesions (LSIL); and high-grade squamous intraepithelial lesions (HSIL). Cervical biopsy specimens were interpreted using the WHO classification of Tumors 2020, which describes: low grade dysplasia (CIN1); and high-grade dysplasia including moderate (CIN2) and severe (CIN3) dysplasia [20].

### Statistical method

Sample size calculation: We expected the sensitivity of colposcopy in isolation to be around 60% to detect high grade lesions. Few articles have evaluated the sensitivity of colposcopy alone, therefore an approximate average of values mentioned in available literature on colposcopy was used [8–13]. A simulation study was conducted to assess the smallest improvement in sensitivity with the cytology for which the statistical power was 90% with the above-mentioned sample size. The simulation study was generated under a two-sided risk alpha of 5% and crossed random effects (standard deviation of 0.40). Ultimately, with the planed sample size, the power is 90% or more to detect an absolute difference in sensitivity of 7% or more (60% for colposcopy in isolation versus 67% for colposcopy and cytology). The power calculation was similar for specificity.

### Statistical analyses

We used mixed effects logistic regression models with two crossed random effects on the intercept: one random effect models the between-readers variability, and one random effect models the between-cases variability. We expect that the sensitivity varied across readers but also across cases as some cases may be more difficult to interpret than others. Statistical analyses were conducted with software R (R Core Team (2022)).

## Results

### Patient characteristics

We included a total number of 80 patients who were referred to our colposcopy clinic. The principal reason for referral was an abnormal pap smear. The mean age of the women included at the time of colposcopy was 33.4 years (standard deviation 7.3 years). Twenty-two women (27.5%) were aged under 30, and 58 (72.5%) were aged over 30. For the 40 patients classified as “<CIN2”, 13 had a normal histology result, 12 had inflammation or metaplasia, and 15 had a CIN1 histology result. Amongst the 40 patients classified as “≥CIN2”, 9 had a CIN2 histology result, 28 had a CIN3 histology result, and 3 had AIS cytology result. Regarding the cytology, 18 had normal or inflammation results, 10 had ASC-US, 18 had LSIL, 15 had ASC-H, 15 had HSIL, 1 had an AGC result and 3 had an AIS result.

### Colposcopist characteristics

The average experience in colposcopy of the participants was 10.9 years with a standard deviation of 9.5 years, and range of 1 to 35 years. Overall, 37,8% (*n* = 14) of colposcopists performed less than 50 colposcopies per year, and 63,2% (*n* = 24) performed more than 50 colposcopies per year. The washout period between the two rounds was 9.4 weeks on average.

### Diagnostic performance: with cytology versus without cytology

**Fig. 2:**
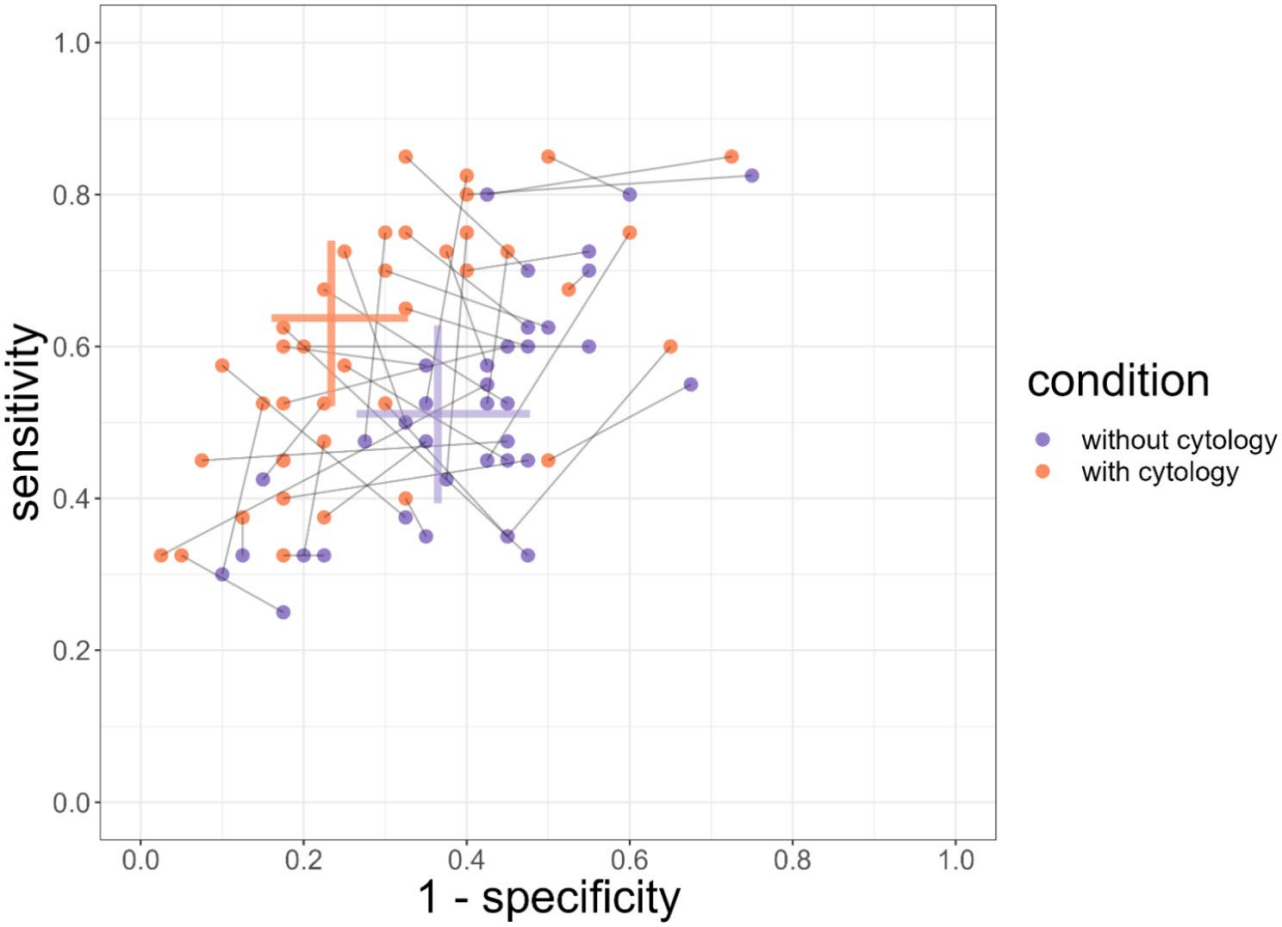
Sensitivity and 1-specificity per rater, with (orange circles) and without (purple circles) cytology. The horizontal (respectively vertical) lines represent the 95% confidence intervals of the overall sensitivities and specificities with and without cytology

The overall sensitivity of colposcopy without cytology was 51.1% [95%CI: 39.3 to 62.8], and 63.7% [95%CI: 52.1 to 73.9] with knowledge of cytology. The presence of cytology increased the chance of correctly identifying a patient as positive with an odds ratio (OR) of 1.68 [95%CI: 1.42 to 1.99]. The overall specificity of colposcopy without cytology was 63.5% [95%CI: 52.3 to 73.5], and 76.7% [95%CI: 67.2 to 84.0] with knowledge of cytology. Knowing the cytological result increased the chance of correctly identifying a patient as negative with an OR of 1.88 [95%CI: 1.57 to 2.24]. This is illustrated in Fig. 2 which represents the overall sensitivity and specificity of each colposcopist in both assessments of the cases.

### Participant’s experience associated with performance

For colposcopists who performed fewer than 50 colposcopies per year, the sensitivity was 55.9% [95%CI: 44.0 to 67.1] without cytology and 66.8% [95%CI: 55.5 to 76.5] with cytology. Having the cytological result available increased the likelihood of correctly identifying a patient as positive, with an OR of 1.59 [95%CI: 1.21 to 2.08]. In this subgroup, the specificity without cytology was 52.1% [95%CI: 41.1 to 62.6], which improved to 72.7% [95%CI: 63.2 to 80.5] with cytology. The presence of cytology increased the chance of correctly identifying a patient as negative, with an OR of 2.45 [95%CI: 1.86 to 3.22]. For colposcopists who performed more than 50 colposcopies per year, the sensitivity without cytology was 48.4% [95%CI: 33.6 to 63.6], and 62.7% [95%CI: 47.5 to 75.8] with cytology. In this group, the inclusion of cytology increased the likelihood of correctly identifying a patient as positive, with an OR of 1.79 [95%CI: 1.44 to 2.23]. The specificity without cytology was 71.9% [95%CI: 57.6 to 82.8], and 80.1% [95%CI: 68.0 to 88.4] with knowledge of cytology. Having the cytological result available increased the chance of correctly identifying a patient as negative, with an OR of 1.57 [95%CI: 1.24 to 1.99].

### Association between colposcopic diagnosis and histology and cytology grades

In Table 1, sensitivity and specificity were assessed in histological sub-groups. Specificity for detection of benign appearances of the cervix was increased by 12.8% when cytology was known. Specificity for detection of CIN1 was increased by 13.8% when cytology was known. For the detection of CIN2 lesions, sensitivity was increased by 17.7%, and for the detection of CIN3 lesions sensitivity was increased by 7.7%. Sensitivity and specificity were also analysed in cytological sub-groups, presented in Table 2. The cytology information communicated to the colposcopists was binarised into ‘benign/borderline’ (normal, inflammation, ASC-US, LSIL) and ‘high-grade’ (ASC-H, HSIL, AGC, AIS). Sensitivity when cytology was reported as high-grade was 75.5% [95%CI: 63.3 to 84.6] compared to 36.9% [95%CI: 21.0 to 56.3] when cytology was reported benign. A high-grade cytology increased the chance of correctly identifying a patient as positive by an OR of 5.25 [95%CI: 2.11 to 13.12]. Specificity when cytology was reported as benign was 81.5% [95%CI: 72.7 to 88.0] compared to 50.5% [95%CI: 28.0 to 72.8] when cytology was reported as high-grade. A benign cytology increased the chance of correctly identifying a patient as negative by an OR of 4.33 [95%CI: 1.54 to 12.14].

**Table 1:** Specificity in patients identified benign/borderline and CIN1 by histology and sensitivity in patients identified as CIN2 and CIN3 by histology in each condition (with and without cytology)

		Without cytology	With cytology	OR [95%CI]
Specificity	Benign	0.651 [0.512 to 0.769]	0.779 [0.663 to 0.863]	1.88 [1.50 to 2.37]
	CIN1	0.610 [0.450 to 0.749]	0.748 [0.607 to 0.850]	1.90 [1.43 to 2.52]
Sensitivity	CIN2	0.206 [0.098 to 0.381]	0.383 [0.209 to 0.593]	2.40 [1.64 to 3.50]
	CIN3	0.621 [0.501 to 0.727]	0.698 [0.586 to 0.791]	1.41 [1.16 to 1.72]

CIN1: cervical intraepithelial neoplasia grade 1; CIN2: cervical intraepithelial neoplasia grade 2; CIN3: cervical intraepithelial neoplasia grade 3; OR: odds ratio

**Table 2:** Sensitivity and specificity for patients identified as benign vs. high-grade by the cytology

		Without cytology	With cytology	OR [95%CI]
Patients with a benign/borderline cytology	Sensitivity	0.598 [0.403 to 0.766]	0.369 [0.210 to 0.563]	0.39 [0.29 to 0.54]
	Specificity	0.636 [0.515 to 0.742]	0.815 [0.727 to 0.880]	2.53 [2.06 to 3.10]
Patients with a high-grade cytology	Sensitivity	0.474 [0.336 to 0.616]	0.755 [0.633 to 0.846]	3.41 [2.75 to 4.24]
	Specificity	0.621 [0.384 to 0.811]	0.505 [0.280 to 0.728]	0.62 [0.42 to 0.92]

OR: odds ratio

## Discussion

Our study was designed to evaluate to what extent knowledge of cytology results affects interpretation of colposcopy. Our main finding supports that a known cytology influences the colposcopist’s diagnosis. Our study showed an improvement in sensitivity for ≥ CIN2 detection when cytology was available (51.1% vs. 63.7%). Information offered by cytology, particularly in the case of women with a high-grade result, may make the colposcopist more attentive to possible cervical abnormalities that would have otherwise been overlooked. This allows disease that would be missed to be correctly identified and may therefore reduce morbidity and mortality by facilitating disease treatment at an early stage. Equally, a benign or borderline cytology increased the chances of correctly identify a patient as negative. The improved specificity demonstrated in this study (63.5% vs. 76.6%) has important implications, namely reducing the chances of women having unnecessary cervical biopsy and prolonged follow-up which may be associated with discomfort, emotional distress, and extra financial costs.

Since the initial cytology had contributed to the diagnostic outcome (< CIN2 and ≥ CIN2), some level of inflation of the contrast between colposcopy interpretation with and without knowledge of cytology cannot be excluded.

The principal strength of our study is the large number of colposcopists who took part, increasing statistical power to elucidate the effect of cytology on the overall performance of colposcopy. Additionally, the use of a crossover design to account for individual variation and possible confounding factors strengthens the validity of the results. Finally, the use of a logistic regression mixed model to calculate sensitivities and specificities also enhances the statistical rigour. A relative weakness of this study is that static images were used which does not completely reflect the process of colposcopy. Static images do not convey the ability of the colposcopist to manipulate the cervix in order to visualise the entire squamocolumnar junction, to identify lesions that may be partially hidden inside the os. Another important difference is that when evaluating static images, the interpreter loses the potential to assess the dynamic acetowhite character of cervical tissue. The dynamic nature of acetowhite changes have been proposed as an important factor in determining the likelihood of ≥ CIN2 pathology [21]. Despite this, studies have shown that sensitivity of diagnosing ≥ CIN2 can be very high even when using solely static images [22].

Considering that cytology results are typically available at the point of colposcopy in high income settings worldwide, there is very little literature examining how cytology influences the colposcopist, and therefore to what extent it is beneficial to know the cytology result during colposcopy. One notable study showed, like us, that ≥ CIN2 lesions were more frequently missed when the referral PAP was negative [15]. However, after analysis, the investigators concluded that this was because these lesions were smaller at colposcopy, and that referral PAP result had no influence when this was taken into account. Our study did not evaluate the effect of the size of the lesion during colposcopy. This study added to existing literature suggesting that, as a tool in isolation, examination of cervical appearances after acetic acid application may have relatively poor sensitivity [23]. Our reported sensitivity of 51% for the detection of ≥ CIN2 implies that there are nearly equal numbers of true positives and false negatives, and therefore colposcopy alone likely represents an insufficient measure when determining whether there is significant pathology present.

An interesting result generated by this study was that healthcare professionals with greater recent experience (> 50 colposcopies per year) had a worse sensitivity for the detection of ≥ CIN2 (48.4% without cytology and 62.7% with cytology) than their colleagues undertaking fewer colposcopies (55.9% without cytology and 66.8% with cytology). They did, however, record a superior specificity. This is consistent with other literature in the field which reports that increasing colposcopic experience does not always equate to better sensitivity for the diagnosis of ≥ CIN2 at colposcopy [24]. The population size of this study could have impacted on the difference in sensitivities and may explain this result. A different interpretation of this could be that senior healthcare workers - who have greater overall experience - may do fewer colposcopies each year than their junior counterparts, as they have more responsibilities outside of the direct clinical environment.

In cervical cytology, also a rather subjective diagnostic activity, the cytologist’s pre-knowledge of HPV status has been reported to influence its accuracy [25]. With screening based on HPV test results becoming commonplace, the role of HPV testing in colposcopy, and its sensitivity and specificity in detecting ≥ CIN2 lesions should be investigated. In regards to our study, HPV data analysis was not conducted. However, the effect of knowing HPV-status on the overall performance of colposcopy, paired with knowledge of cytology or alone, will require investigation and could be conducted in a format similar to this study.

## Conclusions

In conclusion, our findings support that knowledge of cytology results significantly influences the interpretation of colposcopy, leading to improved sensitivity for detecting ≥ CIN2 lesions. In cases of high-grade results, colposcopist performance is improved to a greater degree. This may be attributed to the association between cytology and colposcopy results.

## Data Availability

The datasets used and/or analysed during the current study are available from the corresponding author on reasonable request.
